# Case of Obturator or Thyroidal Hernia Successfully Relieved by Operation

**Published:** 1851-08

**Authors:** Henry Obre

**Affiliations:** Formerly Assistant-surgeon to the St. Marylebone Infirmary


*Case of Obturator or Thyroidal Hernia successfullg relieved by Operation.* By Henry Obre, formerly Assistant-surgeon to the St. Marylebone Infirmary.—After commenting on the extreme rarity of this form of hernia, and stating that he had been unable to find any record of its having been detected and relieved by operation during life, the author relates a case in which he operated successfully. The patient, a female aged fifty-five, the mother of a large family, was seized with symptoms which led her medical attendant (Mr. Gardener) to believe that she was suffering from rupture. She denied that this was the case, and a careful examination convinced Mr. Gardener that there was no hernia in the usual situations of that disorder. A little below the femoral region on the right side, however, he detected a degree of hardness resembling a small gland, and deeply seated; with some general fulness about the part. The author saw this patient on the fourth day after the symptoms had begun; at this time, she was suffering extreme abdominal pain in the umbilical region; during the previous twelve hours, her vomiting had been stercoraceous and incessant; the countenance pale and contracted; voice faltering; pulse, weak, small, and intermitting—in short, all the symptoms of pending dissolution from strangulated intestine were present. On careful examination, nothing could be detected but a slight degree of fulness in Scarpa's triangle on the right side; that on the opposite side being well marked; on using firm pressure with the ends of the fingers over the neighborhood of the femoral artery, and a little below the saphenous opening, a distinct hardness was to be felt, slight in extent, but giving the impression as if the sheath of the vessels was being pressed on. The state of the patient was such as to induce the author to propose to make an incision into the upper part of the thigh, down to the hard structure, in the hope that he might find confined intestine low in the femoral canal. He made a straight incision into Scarpa's triangle, as in the operation for tying the common femoral artery; beginning about three inches below Poupart's ligament. When the cribriform fascia was opened, and the saphenous opening exposed, no hernial sac was found, but the hardened structure could be felt lying deep to the inside of this opening. The dissection was with some difficulty continued downwards; the fascia lata was divided, and the pectinæus muscle exposed. The fibres of this muscle were divided transversely for about an inch and a half or two inches, and a hernial sac was exposed, which rose up into the wound to the size of a pigeon's egg. The finger being passed along the sac, entered the obturator opening. The sac was opened, and the intestine was found to be a portion of the small gut, blue and congested. The opening through which it passed did not tightly enclose its neck, but it was considered prudent slightly to divide the edge. In doing this the saphena vein was wounded, and it was necessary to apply a ligature to its upper part. This was the only ligature required. After the operation, no medicine was given; in the course of the day the bowels acted three times, and in the course of a few days the patient had quite recovered.—*London Lancet, July* 5, 1851.

